# Brain Relatively Inert Network: Taking Adult Attention Deficit Hyperactivity Disorder as an Example

**DOI:** 10.3389/fnins.2021.771947

**Published:** 2021-12-03

**Authors:** Hua Zhang, Weiming Zeng, Jin Deng, Yuhu Shi, Le Zhao, Ying Li

**Affiliations:** ^1^Lab of Digital Image and Intelligent Computation, Shanghai Maritime University, Shanghai, China; ^2^College of Mathematics and Informatics, South China Agricultural University, Guangzhou, China

**Keywords:** activation network, relatively inert network, functional MRI, group ICA, functional connectivity, adult ADHD

## Abstract

Resting-state functional MRI (rs-fMRI) has been increasingly applied in the research of brain cognitive science and psychiatric diseases. However, previous studies only focused on specific activation areas of the brain, and there are few studies on the inactivation areas. This may overlook much information that explains the brain’s cognitive function. In this paper, we propose a relatively inert network (RIN) and try to explore its important role in understanding the cognitive mechanism of the brain and the study of mental diseases, using adult attention deficit hyperactivity disorder (ADHD) as an example. Here, we utilize methods based on group independent component analysis (GICA) and *t*-test to identify RIN and calculate its corresponding time series. Through experiments, alterations in the RIN and the corresponding activation network (AN) in adult ADHD patients are observed. And compared with those in the left brain, the activation changes in the right brain are greater. Further, when the RIN functional connectivity is introduced as a feature to classify adult ADHD patients from healthy controls (HCs), the classification accuracy rate is 12% higher than that of the original functional connectivity feature. This was also verified by testing on an independent public dataset. These findings confirm that the RIN of the brain contains much information that will probably be neglected. Moreover, this research provides an effective new means of exploring the information integration between brain regions and the diagnosis of mental illness.

## Introduction

The identification of resting-state networks (RSNs) in the brain from resting-state functional MRI (rs-fMRI) time series (TC) and the analysis of functional connectivity have revealed much about the macroscopic spatio-temporal organization of the brain. The brain’s acquisition of information and decision-making is a manifestation of a range of neural activities. These processes involve a wide range of functional brain networks. Activation responses of voxels are often associated with the multifunctional collaboration of the brain. So even in the same network, different regions may show different levels of activation, and different activation levels can reflect different responses of neurons to different tasks. However, in previous studies, more have been conducted on activated areas of the brain (activation values above a certain threshold). Few have researched relatively inert regions of the brain, ignoring important information about the presence of relatively inert areas. Therefore, this prompted us to study it and try to discover its important role in the discovery of biomarkers of mental illness through this study.

In this paper, we define relatively inactive regions in the RSN as relatively inert networks (RINs), and we explore the important role of RINs in brain cognition in terms of network and functional connectivity. The method of brain activation detection includes correlation analysis (Bandettini, 2010), general linear model (Friston et al., 2010), cluster analysis (Smolders et al., 2007; Slavakis et al., 2018), principal component analysis (PCA) (Andersen et al., 1999; Smith et al., 2014), and independent component (IC) analysis (ICA) (Mckeown et al., 1998; Lin et al., 2010; Du and Fan, 2013; De Blasi et al., 2020). Among them, correlation analysis and general linear models require a prior given stimulus function, so they are more suitable for data analysis of task fMRI. For resting-state data, the detection of activation is equivalent to the identification of RSN. ICA is one of the most widely used methods for estimating brain functional networks. And it is suitable not only for task-state data but also for the study of resting-state data without the need for *a priori* knowledge. It models fMRI data as linear combinations of independent sources and can identify cross-individual patterns and common cohesive components. Since ICA generates ICs in any order, to make ICs comparable across subjects, we used the group ICA (GICA) method to identify RSNs in healthy subjects and attention deficit hyperactivity disorder (ADHD) patients in this study. We stitched fMRI data along the time dimension and implemented ICA on group data; then we used the group information-guided ICA (GIG-ICA) method (Du and Fan, 2013) to obtain group-level ICs; and subject-level ICs were obtained from the group-level ICs. *T*-test-based methods were then used to identify RINs and the corresponding activation networks (ANs). To better mine the information in RINs, this paper provides a method to estimate RINs and AN TC.

ADHD is a neuropsychiatric disorder that affects young children and adolescents. However, ADHD is not a child-specific disorder; it is a chronic process with symptoms that last until adulthood, including remission cases, by up to 50% (Shaw et al., 2013; Rovira et al., 2020). The common behavioral manifestations of ADHD are attention deficit, hyperactivity, and impulsivity (Schneider et al., 2006; Spencer et al., 2007; Roshannia et al., 2021). ADHD patients usually present with a variety of concomitant disorders, and they are overlain by one or more concomitant psychological disorders, such as addiction, borderline disorder, depression, bipolar disorder, and anxiety disorders (Ashcroft et al., 2015; Schiweck et al., 2021). Due to misunderstandings and lack of knowledge about this disease, many people with ADHD are misdiagnosed and often fail to receive effective treatment (Kooij et al., 2010). At the same time, the falsifiability based on the diagnostic scales is also very worrying (Smith et al., 2017; Becke et al., 2021). Therefore, the diagnosis of adult ADHD is more difficult compared with childhood ADHD. Studying structural and functional changes in adult ADHD and further investigating potential biomarkers related to their neural mechanisms are essential for their early detection and more effective treatment (dos Santos Siqueira et al., 2014; Kessler et al., 2014; Buitelaar et al., 2021; Duan et al., 2021; Versace et al., 2021). In recent years, rs-fMRI has been enhanced in the field of ADHD and has acquired many influential neurocognitive networks (Beckmann et al., 2005; Power et al., 2011). Previous studies have identified changes in functional connectivity between the default mode network (DMN) and networks that support attention and cognitive control in ADHD patients (Sudre et al., 2017), and abnormal network connectivity is related to the severity of symptoms (Qian et al., 2019). However, it is unclear whether these also appear in RINs and whether abnormal changes occur in RINs.

**FIGURE 1:**
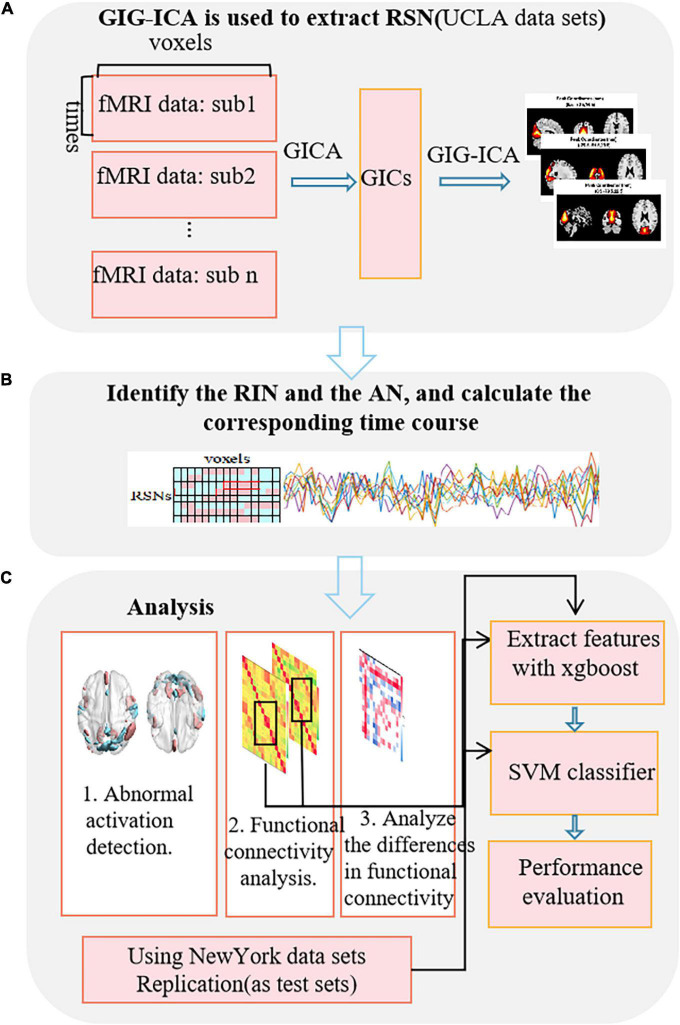
Flowchart of the research method. **(A)** Extraction of the RSN using GIG-ICA. **(B)** Identification of the RINs and the ANs, and calculation of the corresponding time-series. **(C)** The analysis process based on RINs and ANs.

This paper is the first to study the RIN of the brain and to use adult ADHD data as an example to explore its application in the research and diagnosis of mental diseases. Figure 1 shows the entire research process. In the first step (Figure 1A), the GICA method is used to obtain RSN, and then it is selected. In the second step (Figure 1B), the RIN and AN were identified, and then the corresponding TC were estimated using our proposed method. Finally, (Figure 1C) the extracted features were analyzed and used for the training and testing of the support vector machine (SVM) classifier. We observed that RIN and AN were significantly different between adult ADHD patients and healthy controls (HCs). Also, we compared the differences in functional network connectivity (FNC) between the two groups of subjects. In addition, the identification of adult ADHD patients from HCs by a model based on FNCs with RINs and ANs was significantly better than that by a model characterized by original functional connectivity. This was also verified based on independent dataset testing. These suggest that the RIN also plays an important role in brain information transmission.

## Materials and Methods

### Independent Component Analysis

The ICA method assumes that the observed random signal *X* obeys the model as follows (Mckeown et al., 1998):


(1) X=A⁢S

Since the number of voxels in spatio-temporal fMRI data is much more than the number of time points, the spatial ICA method is usually more suitable for modeling given fMRI data. *X* = (*x*_1_, *x*_2_,… *x*_*t*_, *… x_*k*_*)*^T^* represents a random variable in the spatial ICA, *K* is the number of time points, *x*_*t*_ is a vector of length *L*, and *X* represents a given 3D fMRI image with L voxels at time *K*. *S* = (*S*_1_, *S*_2_,… *S*_*N*_)*^T^* is *N* unknown and mutually independent source signals (corresponding to the function network), *A* is an unknown mixing matrix, and the purpose of ICA is to estimate the mixing matrix *A* and the source signal *S* only through *X*. The ICA model has two assumptions about the source signal: one is that the observed signal is a linear combination of several statistical independent source signals and the other is that the distribution of the source signal is non-Gaussian (at most one can be Gaussian).

Since both *A* and *S* are unknown, their exact values cannot be calculated in the actual solution. The separation matrix *W* and the estimate *Y* can be estimated by optimizing the independence of *S*. For example, the classic ICA method uses minimizing mutual information (Amari et al., 1996), and Hyvarinen used maximizing non-Gaussian (measured by negentropy or kurtosis) to estimate these components (Hyvarinen, 1999). Specifically, it finds a separation matrix W by constantly updates and iterations:


(2) $$Y=\left(W\,A\right)\,S=\tilde{S}$$

When *W* is closer to *A*^–1^, *Y* is closer to *S*.

### Group Independent Component Analysis

An effective method of multi-subject analysis is GICA (Allen et al., 2012), which can identify ICs at the group level and reconstruct the independent specific subject components at the subject level. Compared with ICA for each subject separately, it is easier for GICA to establish a direct correspondence of different subject ICs. First, the group IC (GIC) is calculated by the GICA method, and then specific subject ICs are reconstructed by using the GIC. Common reconstruction methods include GICA (Chen et al., 2008), constrained ICA (cICA) (Wei and Rajapakse, 2005), spatio-temporal regression (Nickerson et al., 2017), and GIG-ICA (Du and Fan, 2013). In this paper, the GIG-ICA is used to extract brain functional networks in fMRI data, and specifically, the method transformed this process into the multi-objective optimization problem described in (3).


(3) $$\begin{array}{c} \max\,\left\{ \begin{array}{c} Q\,\left(w_{i}\right)={\left\{E\,\left[G\,\left(Y_{i}\right)\right]-E\,\left[G\,\left(z\right)\right]\right\}}^{2}\\ C\,\left(w_{i}\right)=\phi \,\left\{Y_{i},R_{i}\right\} \end{array}\right.\\ s.t.∥w_{i}∥=1 \end{array}$$

*Q*(*w*_*i*_) is the negative entropy of *Y*_*i*_, which is the *i*th estimated IC, $Y_{i}=w_{i}^{T}\,\tilde{X}$; $\tilde{X}$ is the random vector after PCA whitening, and *R*_*i*_ represents the spatial reference that is the GIC of the zero mean and unit variance in the previous step after normalization. *E*[.]represents a mathematical expectation, *G*[.]is an arbitrary non-quadratic function, and *z* is a Gaussian variable with zero mean unit variance. *C*(*w*_*i*_), which is specifically defined as ϕ{*Y*_*i*_, *R*_*i*_}=E[*Y*_*i*_ *R*_*i*_], is used to measure the closeness between the estimated component *Y*_*i*_ and the reference *R*_*i*_. By solving the multi-objective optimization problem, the optimal separation column vector *w*_*i*_ is generated to maximize the independence of *Y*_*i*_ and maximize the proximity of the reference *R*_*i*_ and the independence of *Y*_*i*_ at the same time. To solve the multi-objective optimization problem, it is usually necessary to find the Pareto optimal set or its subset and to strictly evaluate which specific compromise solution is more suitable for the researched problem. To prevent the optimization from being dominated by a larger cost function, the *arctan* function is used to normalize *Q*(*w*_*i*_), and the objective function is transformed into the following formula:


(4) wi*=arg⁢maxw⁢i⁡F⁢(wi)=arg⁢maxw⁢i⁡[a⋅K⁢(wi)+(1-a)⋅C⁢(wi)]s.t.∥wi∥=1    0<a<1

Here, *a* is the weighting parameter, which can be determined empirically. Use the gradient descent method to continuously iterate for solving *w*_*i*_, and calculate *Y*_*i*_. The corresponding TC *T*_*i*_ is equivalent to the mean of blood oxygenation level-dependent (BOLD) series of all voxels weighted by its associated IC z-score.

### Identification of Relatively Inert Network and Activation Network

During a resting-state scan, the internal network patterns of the brain may change substantially (Chen et al., 2015). If we focus only on the activated areas of the brain, it is easy to overlook the potential information in relatively inert areas. We used the GIG-ICA method to separate the signals from the rs-fMRI data of HCs, and we processed the individual components with a one-sample *t*-test. We defined the area higher than the threshold μ in each IC (corresponding to a certain RSN) as AN and the other area as RIN. Thus, for each RSN, the corresponding AN and RIN can be further obtained. On this foundation, we propose the following method to estimate the TC corresponding to each AN and RIN, and the sum of their TC is the same as the TC of that original IC.


(5) $$T_{a\,c\,t_{i}}=\frac{1}{V\,a_{i}}\,\sum_{v=1}^{v}\left(y_{i\,v}\cdot \,d_{v}\cdot f_{v}\right)$$

(6) $$T_{i\,n\,e_{i}}=\frac{1}{V\,e_{i}}\,\sum_{v=1}^{v}\left(y_{i\,v}\cdot \,\left|1-d_{v}\right|\cdot f_{v}\right)$$

*T*_*act*_*i*__ represents the TC corresponding to the AN, and *T*_*ine*_*i*__ represents the TC corresponding to the RIN. *Ve*_*i*_ and *Va*_*i*_ represent the number of voxels in the RIN and AN of the *i*th component, respectively. *V* is the total number of voxels, *f*_*v*_ is the *v*th column of *X*, and *y*_*iv*_ is the *v*th value of the *i*th component. *d*_*v*_ is the *v*th value of *d*(*V*×1), which indicates whether the *v*th voxel is activated, 1 indicates activated, and 0 indicates inactive. So the estimation is equivalent to the mean of BOLD series of the corresponding areas voxels weighted by the z-score of their associated IC.

## Experiments and Results

### Materials and Data Preprocessing

The neuroimaging dataset shared by the University of California, Los Angeles (UCLA) Neuropsychiatric Research Association (Poldrack et al., 2016), which includes 138 healthy subjects, 58 subjects with schizophrenia, 49 subjects with bipolar disorder, and 45 subjects with ADHD, was downloaded. The dataset is shared through the OpenfMRI project and formatted according to the brain imaging data structure (BIDS) standard. The participants were asked to stay relaxed and to keep their eyes open, without giving them any stimuli or asking them to respond, and then were scanned to obtain resting-state data using the 3T Siemens Trio scanner (Siemens Healthineers, Erlangen, Germany). A total of 152 time points were scanned. T2*-weighted echo-planar imaging (EPI) sequence was used to collect functional MRI data with the following parameters: repetition time (TR) = 2 s, echo time (TE) = 30 ms, flip angle (FA) = 90°, field of view (FOV) = 192 mm × 192 mm (64 × 64 matrix), number of slices 34, and slice thickness = 4 mm. For more information about the dataset, please refer to the link: https://openneuro.org/datasets/ds000030. The resting-state data of healthy and ADHD subjects aged 21--50 years were used in this paper. The SPM12 software^1^ was used to preprocess the data including removal of the first 10 time point images, slice timing correction, motion correction, spatial standardization using the Montreal Neurological Institute (MNI) EPI template, and use of 4-mm full width at half maximum (FWHM) Gaussian kernel to smooth in space. Subjects with excessive motion were excluded, and 42 healthy and 41 ADHD subjects with matching gender and age were selected.

**FIGURE 2:**
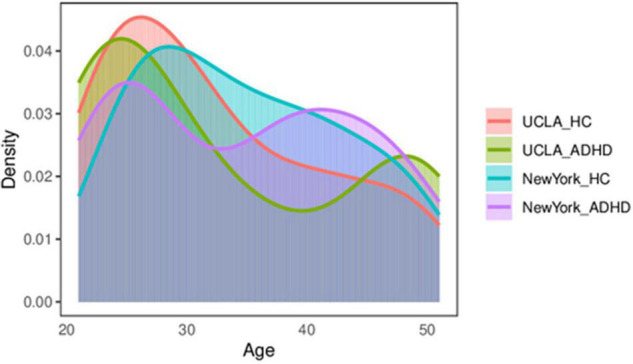
The distribution of the control–ADHD population. ADHD, attention deficit hyperactivity disorder.

To assess the robustness of our results, our method was replicated on an independent publicly ADHD dataset, publicly available for download at http://fcon_1000.projects.nitrc.org/fcpClassic/FcpTable.html. This dataset has 25 ADHD patients and 84 healthy subjects. These subjects were recruited by the adult ADHD program group at the New York University School of Medicine, United States. The subjects laid flat in the MRI machine with their eyes open and in a relaxed awake state. The scan TR = 2 s, TE = 25 ms, scan resolution was 64 × 64, intra-slice resolution was 3 mm × 3 mm, slice thickness = 3 mm, and number of slices was 39, covering the entire brain area; and a total of 197 time points were scanned. Subjects who did not undergo psychiatric evaluation or were younger than 20 were excluded. The same preprocessing method used for the UCLA data was used, and subjects with excessive head movements were excluded. To avoid a confounding effect due to an imbalance in the sample, 23 healthy subjects and 24 patients with ADHD were finally selected. The two groups of subjects were matched in terms of gender and age. All subjects were between 20 and 50 years of age. The distribution of the HC-ADHD population for these two datasets is shown in Figure 2. Table 1 shows the age and gender indices of the two datasets. According to the results of statistical tests, there were no significant differences in age and gender between the two groups for each dataset.

**TABLE 1:** Age and gender indices of two independent datasets.

	UCLA		New York	
	HC	ADHD	HC	ADHD
Number of participants	42	41	23	24
Age (years)	32.45 ± 9.05	32.85 ± 10.89	34.89 ± 8.387	34.75 ± 9.712
Gender (M/F)	21/21	20/21	14/9	19/5
Statistical test (*p*-value): age/gender	0.85/0.91		0.96 (*t*-test)/0.19 (chi-square test)	

*UCLA, University of California, Los Angeles; HC, healthy control; ADHD, attention deficit hyperactivity disorder.*

### Group Independent Component Analysis and Component Identification

**FIGURE 3:**
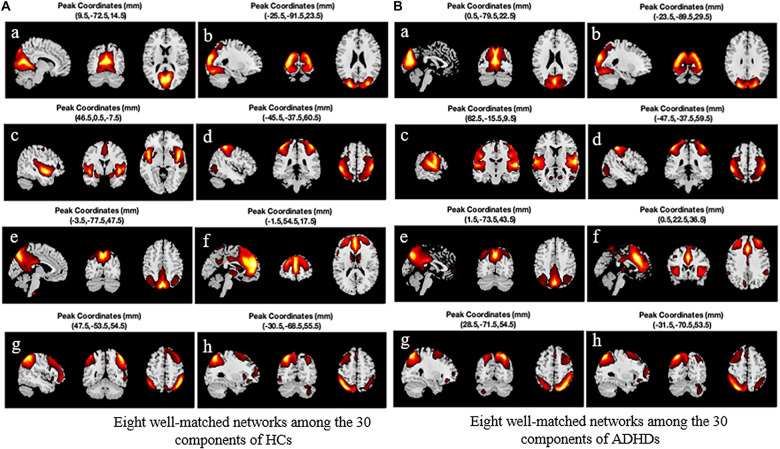
Eight best-matched intrinsic connectivity networks (ICNs) and their peak activation coordinates. **(A)** The eight best networks among the 30 components extracted from HC. **(B)** The eight best networks among the 29 components extracted from ADHD. HC, healthy control; ADHD, attention deficit hyperactivity disorder.

The PCA is used to reduce the dimensionality of each subject’s data for simplifying the ICA algorithm. Then the time cascade method is used to combine all the subjects’ reduced data. Another PCA (group level) was performed to further reduce the time dimension of the group data to the number of IC, and then the Infomax algorithm (Bell and Sejnowski, 1995) was applied to extract the GIC. GIFT software (version 3.0c)^2^ was used to perform GICA in the two groups of subjects. Meanwhile, the minimum description length (MDL) standard (Li et al., 2007) was utilized to estimate the dimensions to determine the number of components. HCs were estimated with 30 components, and ADHD with 29. In order to obtain a reliable GIC, the ICASSO method (Himberg and Hyvarinen, 2003) was used to repeat the ICA 20 times. And the GIG-ICA method was used to reconstruct the corresponding TC and the unique IC of each subject. The IC that we needed was identified and picked out. In the first step, the correlation between the spatial map of each component and the prior mask map of gray matter, white matter, and cerebrospinal fluid (CSF) provided in the DPABI software package (Yan et al., 2016) was calculated in the MNI standardized brain space. Since components with high correlation with prior CSF or white matter or low correlation with the gray matter may be affected by human factors (Assaf et al., 2010), they were excluded to ensure that the selected components are mainly concentrated in the gray matter area. In the second step, due to the low-frequency characteristics of the BOLD signal, the power spectrum of the network should show higher low-frequency spectrum power. Therefore, the ratio of the low-frequency power to the high-frequency power of each component was calculated, and the component with a higher ratio was screened out (most ratios of low-frequency to high-frequency power of some networks are greater than 10). The third step is to calculate the spatial correlation between the remaining components and the eight core intrinsic connectivity networks (ICNs) reported by Beckmann et al. (2005) and to select the eight best-matched components with the visual inspection and correlation coefficients. These eight networks are (a) medial visual network (MVN), (b) lateral occipital visual cortex (LO), (c) auditory area network (auditory), (d) sensorimotor area (sensorimotor), (e) DMN, (f) executive control network (ECN), and (g) and (h) right-lateralized fronto-parietal network (RFPN) and left-lateralized fronto-parietal network (LFPN). Figure 3 shows the sagittal, coronal, and cross-sectional planes of the eight best-matched ICNs. Figure 3A (left) shows the activation peak coordinates of eight ICNs from a to h in the HC group, and Figure 3B (right) shows the activation peak coordinates of 8 ICNs from a to h in the ADHD group. It is worth noting that after comparing the activation peak coordinates of the eight large-scale networks of the two groups of subjects, it is found that the network with the closest peak coordinates in the sensorimotor area is followed by LO, LFPN, and DMN, while the network with the largest difference in activation peak coordinates in the auditory area is followed by RFPN and ECN.

### Comparing Group Differences in the Relatively Inert Networks and the Activation Networks

**FIGURE 4:**
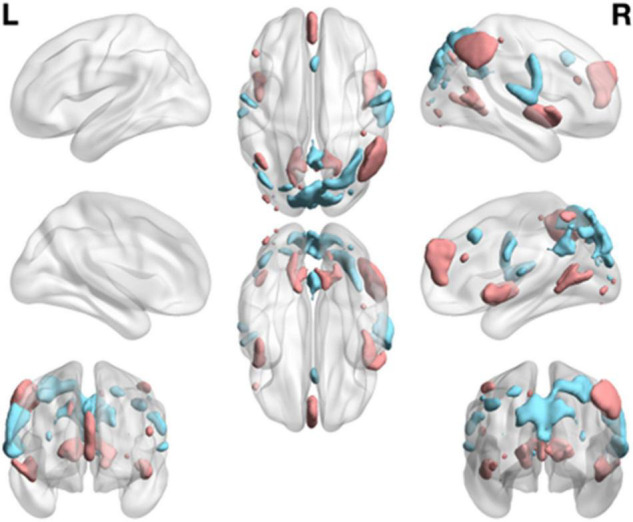
Group differences of functional network maps between ADHD patients and HCs. It is a summary of eight functional network differences. The intersection of the RIN of the HCs and the AN of the adult ADHD; and the areas that satisfy the two-sample *t*-test and have significant differences are shown as cold areas. The intersection of the AN of HCs and the RIN of adult ADHD; and the areas that satisfy the significant difference of the two-sample *t*-test are shown as hot area. Use thresholds of *p* < 0.05 and μ = 3 to display clusters. L, left hemisphere; R, right hemisphere. HC, healthy control; ADHD, attention deficit hyperactivity disorder; RIN, relatively inert network; AN, activation network.

A one-sample *t*-test was used to determine the RINS and ANS of HCs and ADHD patients. In this way, each RSN could be divided into AN and RIN. The areas with different activation levels in the two groups of subjects were counted, and a two-sample *t*-test was further performed. Figure 4 shows the areas where ANs and RINs differed significantly between the two groups of subjects when the threshold μ = 3 (*p* < 0.05). There are two scenarios for this difference. One is that the areas (indicated by cold areas) are the intersections of the RINs of HCs and the ANs of adult ADHD; i.e., HCs show no activation, whereas adult ADHD patients show significant activation. These areas show abnormally enhanced activation in adult ADHD patients; the second (indicated by hot area) is that the areas are the intersections of ANs of HCs and RINs of adult ADHD; i.e., HCs show activation, but ADHD patients show no activation. In other words, the activation in these areas is significantly weakened in adults with ADHD. Some responses in cognitive function are usually reflected in the brain activation. Statistical analyses were conducted in these abnormal areas. Compared with those in HCs, the areas of enhanced activation in adult ADHD patients were concentrated in the precentral gyrus (Brodmann areas 4 and 6), insula (Brodmann area 13), postcentral gyrus (Brodmann areas 3, 43, and 40), cuneus (Brodmann areas 7, 18, and 19), and superior temporal gyrus (Brodmann area 41). The areas of weakened activation were mainly concentrated in the insula (Brodmann area 13), superior temporal gyrus (Brodmann area 22), medial frontal gyrus (Brodmann areas 8, 9, and 10), and inferior parietal lobule (Brodmann areas 7, 39, and 40). Furthermore, when analyzing RSN activation in both groups of subjects, it was found that the activation difference of adult ADHD patients is distributed in both the left and right hemispheres, compared with that of the HCs. However, Figure 4 shows that there are more areas of discrepancy in the right hemisphere than in the left hemisphere.

### Group Differences in Functional Network Connectively

**FIGURE 5:**
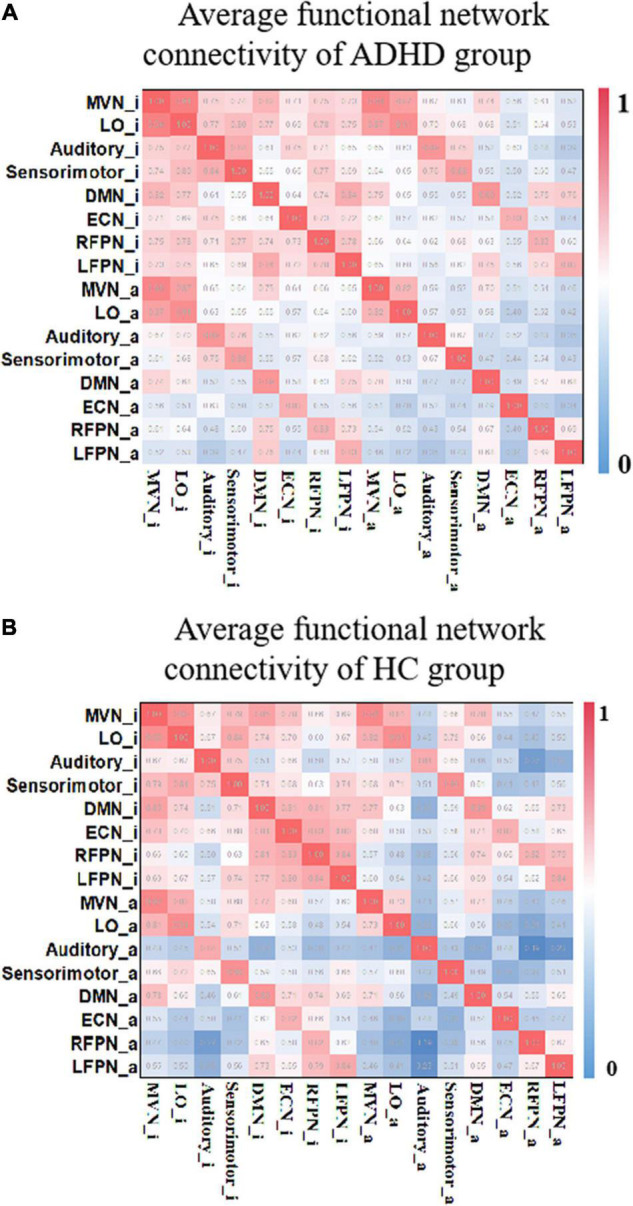
The average functional connectivity of each group of subjects. **(A)** The FNCs of the HC group. **(B)** The FNCs of the ADHD group. “_i” represents the corresponding RIN, and “_a” indicates the corresponding AN. Both include the RIN and the AN, and μ = 3. FNC, functional network connectivity; HC, healthy control; ADHD, attention deficit hyperactivity disorder; RIN, relatively inert network; AN, activation network.

The FNC reflects, to some extent, the information interaction between different networks (Power et al., 2011). In this study, the method in section “Identification of Relatively Inert Network and Activation Network” was used to estimate the TC corresponding to the RINs and ANs for each participant. Applying Pearson’s correlation coefficient to calculate the FNC, a 16 × 16 FNC matrix was obtained for each subject. Figure 5 shows the mean FNC for each group of subjects. The bottom left corner of the matrix shows that there is some connectivity between RINs and ANs. Also, comparing Figures 5A,B, it was observed that AN and RIN of the same functional network are highly connected for both ADHD patients and HCs. And the connectivity between RINs is higher than the connectivity between ANs. In addition, the differences in functional connectivity between different networks were more pronounced in HCs than in ADHD patients.

**FIGURE 6:**
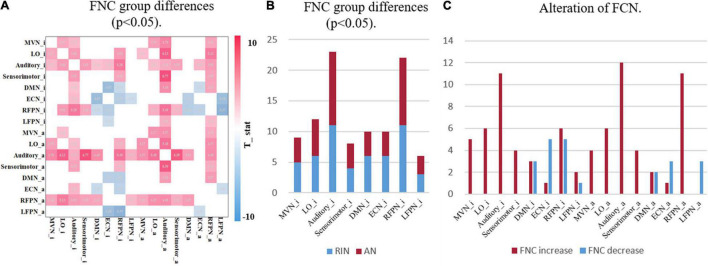
Group differences in functional network connectively. **(A)** The FNC with significant differences between HCs and ADHD patients under the two-sample *t*-test. Only the case of *p* < 0.05 (FDR corrected) is shown here, where red indicates that the FNC of ADHD patients is stronger than that of HCs, and blue indicates that the FNC of ADHD patients is lower than that of HCs. **(B)** The statistical result of a significant alteration in FNC. The horizontal axis is the functional network, and the vertical axis is the number of alterations FNCs. RIN, relatively inert network; AN, activation network. **(C)** The statistics of enhanced and weakened network connectivity of FNC. Both with μ = 3. FNC, functional network connectivity; HC, healthy control; ADHD, attention deficit hyperactivity disorder; FDR, false discovery rate.

Accurate identification of disease-specific induced changes in functional connectivity is considered an important task that can highlight the underlying mechanisms of the disease. Therefore, a two-sample *t*-test was performed for each element of the FNC matrix to compare the different functional connectivity between the two groups. Figure 6A shows the FNCs with significant differences between HCs and adult ADHD [μ = 3, *p* < 0.05, false discovery rate (FDR) corrected]. Figure 6B shows the number of altered connections for each network. It can be found that the auditory area and RFPN changed the most. Also, the enhancement and weakening of the FCN were further counted. Figure 6C shows that the connectivity of ECN_i was significantly weakened in the ADHD group compared with the HC group. Meanwhile, compared with HCs, adult ADHD patients show a very strong connection in most changed FNCs, especially those involving the auditory area.

### Classification Based on Relatively Inert Networks and Activation Networks

#### Classification Within University of California, Los Angeles, Dataset

**FIGURE 7:**
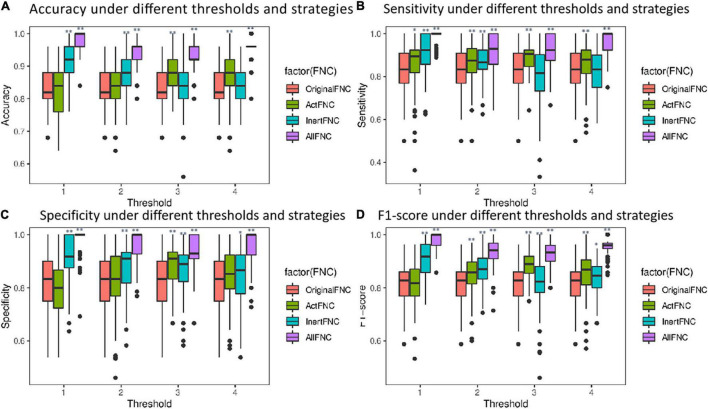
Box plots comparing the performance of classification using different features. Red represents the OriginalFNC, green represents the ActFNC, blue represents the InertFNC, and purple represents the AllFNC. **(A–D)** The horizontal axis is the threshold μ; vertical axis represents the accuracy, sensitivity, specificity, and F1-score. * indicates *p* < 0.05, and ^**^ indicates *p* < 0.01.

In order to verify the importance of RINs in assisting the diagnosis of mental disorders, four different strategies were used to construct features, namely, the connectivity of the original functional network (OriginalFNC), the functional connectivity of ANs (ActFNC), the functional connectivity of RINs (InertFNC), and the functional connectivity of both ANs and RINs (AllFNC). SVM models were utilized to classify HC and ADHD. Thirty percent of the UCLA dataset was used as the test set, and the remaining part as the training set. Feature selection using XGBoost (Chen and Guestrin, 2016) and 10-fold cross-validation was used to train the models. The predicted values of each strategy were obtained for McNemar’s test. As a result, the AllFNC classification performance was significantly higher than the OriginalFNC at μ = 1, 2, and 3 (*p* < 0.05). Further, the data label was randomly shuffled, and the classification was repeated 100 times to test the significance along the massive permutation. Figure 7 shows the box plots of accuracy, sensitivity, specificity, and F1-score for 100 classifications of the four features with different values of the threshold μ.

When μ = 2, AllFNC offers the highest classification (the average accuracy = 94.2%, sensitivity = 92.1%, specificity = 96.3%, and F1-score = 93.7%). The next highest is InertFNC (accuracy = 87.1%, sensitivity = 86.8%, specificity = 87.4%, F1-score = 86.6%) and then OriginalFNC classification (the average accuracy = 82.0%, sensitivity = 82.3%, specificity = 82.1%, F1-score = 81.5%). A two-sample *t*-test was used, and the results showed that both AllFNC and InertFNC were significantly better classified than the OriginalFNC (*p* < 0.01). Similar results can be obtained when μ takes other values.

As with most psychiatric disorders, there are no specific physiological indicators to diagnose adult ADHD (Cortese et al., 2018). To locate mutations more accurately, a single FNC as a feature was used to classify adults with ADHD and HCs, and the experiment was repeated 20 times. Features with F1-scores higher than 0.65 were selected under four thresholds (μ). Table 2 shows the mean of the classification performance of the four best-performing features that were finally screened. When they were examined, it was found that all four features were significantly different, and all showed enhanced ADHD functional connectivity. The classification results of the four best-performing features are shown in Table 3. Notably, all four features were associated with the Auditory_a network. The FNC between Auditory_a and Sensorimotor_i was used as a feature for classification with an accuracy of 0.87 and an area under the curve (AUC) of 0.91.

**TABLE 2:** The comprehensive results of the classification performance for a single feature.

FNC	Mean F1-score
RFPN_i Auditory_i	0.69
RFPN_a-Auditory_i	0.72
RFPN_i-Sensorimotor_i	0.72
RFPN_i-Auditory_a	0.74

*FNC, functional network connectivity.*

**TABLE 3:** Best classification performance of a single feature.

FNC	Mean F1-score
RFPN_i Auditory_i	0.69
RFPN_a-Auditory_i	0.72
RFPN_i-Sensorimotor_i	0.72
RFPN_i-Auditory_a	0.74

*FNC, functional network connectivity.*

#### Generalization to New York Dataset

**FIGURE 8:**
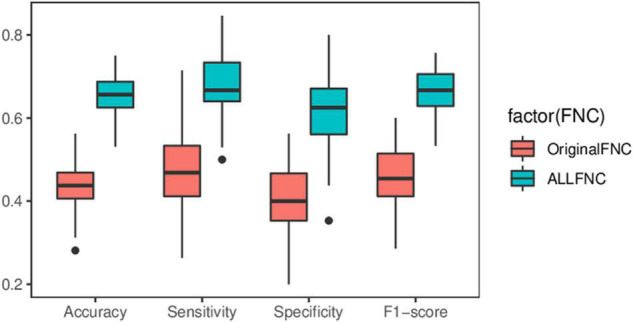
Box plot of predicted performance using independent datasets. Two-sample *t*-test showing significant differences in all indicators.

Sample size has a profound impact on the variability of accuracy and accuracy estimates (Flint et al., 2021). To avoid exaggeration of statistical power and classification parameters, independent publicly available datasets were used to compare the performance of the original functional connectivity and the functional connectivity with the introduction of RINs and ANs on the classification of adult ADHD. Specifically, with the complete UCLA dataset was trained and tested in New York dataset, which features different acquisition parameters. It is worth noting that in practical application scenarios, the labels of the samples are not available before classification. Therefore, for all test sets, cICA was utilized to compute a specific set of eight RSNs, which avoids the restriction in GICA that requires grouping the data in advance. The computation of features in the UCLA data is then replicated. Similarly, the XGBoost method is employed to select the features. Meanwhile, the data were randomly shuffle, SVM was repeated 100 times for the classification results shown in Figure 8. The experimental results show that the average classification accuracy, sensitivity, specificity, and F1-score of the OriginalFNC100 are 0.438, 0.472, 0.402, and 0.459, respectively, while the average accuracy, sensitivity, specificity, and F1-score of the AllFNC100 classifications with the introduction of RIN and AN are 0.647, 0.675, 0.620, and 0.663, respectively. A two-sample *t*-test was further performed on the results of the two methods, and it was found that their differences were significant (*p* < 0.01). Although it is difficult to avoid the degradation of classification accuracy in the independent dataset because more work related to feature engineering was not performed (using only functional connectivity), our method still has a greater advantage over the OriginalFNC.

#### Threshold Analysis

**FIGURE 9:**
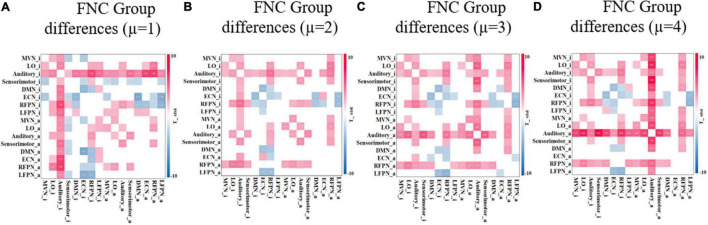
FNC with significant difference between ADHD and HC groups under different threshold μ. **(A–D)** Different thresholds and the two-sample *t*-test *p* < 0.05 (FDR corrected). Red represents that the functional connectivity of ADHD is stronger than that of HC, and blue represents that the functional connectivity of ADHD is weaker than that of HC. FNC, functional network connectivity; ADHD, attention deficit hyperactivity disorder; HC, healthy control; FDR, false discovery rate.

After extraction of the IC (corresponding to the functional network of the resting brain) using the ICA method, the results are usually Z-transformed. For the determination of brain activation areas, the area above the threshold is AN, while the area below the threshold is RIN. The threshold μ can be determined empirically. To assess the effect of the threshold μ on the study results, the experiments compared four different FNCs under different threshold conditions. As the threshold value increases, the range of AN becomes smaller. Therefore, the threshold value is usually not set too large. In this study, ECN_i is empty when μ is not less than 4. Therefore, the differences in FNCs between the two groups of subjects were compared under four conditions with threshold μ equal to 1, 2, 3, and 4. Figure 9 shows the FNC with significant differences between the adult ADHD and HC groups at different threshold value μ. The results show that when μ is taken at different values, there is only a small change in functional connectivity with significant differences between the two groups of subjects. This also suggests that the changes in FNC differences between the adult ADHD and HC groups are within acceptable limits when the thresholds are within a certain range.

To further exclude the effect of different thresholds on the classification performance of HCs and ADHD patients, the classification accuracy, sensitivity, specificity, and F1-score were calculated when the threshold μ was taken with four different values of 1, 2, 3, and 4 in the experiments in section “Classification Within University of California, Los Angeles, Dataset.” As shown in Figure 7, the classification performance using AllFNC is always the best when μ takes different values. Also, the performance of classification using only the functional connectivity features between RINs in most situations is higher than the performance of the original network functional connectivity. Table 4 shows the average accuracy, sensitivity, specificity, and F1-score values for 100 classifications at different threshold values (μ). It can be demonstrated above that there is no significant effect on the classification results when the threshold value is 1–4.

**TABLE 4:** Classification performance of different features under different thresholds.

	Feature	Mean accuracy	Mean sensitivity	Mean specificity	Mean F1-score
-	OriginalFNC	0.820	0.823	0.821	0.815
μ = 1	ActFNC	0.820	0.855	0.795	0.815
	InertFNC	0.910	0.916	0.909	0.908
	AllFNC	0.979	0.981	0.979	0.979
μ = 2	ActFNC	0.849	0.874	0.826	0.848
	InertFNC	0.871	0.868	0.874	0.866
	AllFNC	0.942	0.921	0.963	0.937
μ = 3	ActFNC	0.886	0.891	0.882	0.886
	InertFNC	0.833	0.802	0.873	0.820
	AllFNC	0.930	0.925	0.935	0.928
μ = 4	ActFNC	0.853	0.860	0.850	0.849
	InertFNC	0.838	0.820	0.856	0.832
	AllFNC	0.957	0.960	0.951	0.956
					

## Discussion

In this paper, we propose a method for the identification and estimation of RINs and apply it to the study and diagnosis of the disease using fMRI data of adult ADHD as an example. Compared with the traditional methods, our method focuses not only on the activated areas of the brain but also on the relatively inert areas of the brain. The activated areas of the brain are usually delineated by setting a threshold value. However, brain areas encode information in multivariate responses, and neural computations are nonlinear (Cannon et al., 2011). Strictly speaking, the portion below the threshold (the relatively inert area) is not completely inactive, and weaker or nonlinear reactions may be present. It is likely that these responses are not decomposed by ICA (or other methods of matrix decomposition), since these methods usually assume that the obtained BOLD signal is a linear accumulation of the source signal. As a result, we believe that the relatively inert areas also contain important information that can be used for disease diagnosis.

Specifically, by comparing ANs and RINs, we identified abnormal activation areas in the brains of adults with ADHD compared with healthy subjects. Mainly, the activation was significantly enhanced in the precentral gyrus (Brodmann areas 4 and 6), postcentral gyrus (Brodmann areas 3, 40, and 43), and superior temporal gyrus (Brodmann area 41), while the activation was significantly weakened in the superior temporal gyrus (Brodmann area 22), medial frontal gyrus (Brodmann areas 8, 9, and 10), insula (Brodmann area 13), and cuneus and inferior parietal lobule (Brodmann areas 7, 39, and 40). This suggests that the increased activation in Brodmann areas 4, 6, and 40 in children with ADHD (Suskauer et al., 2008) did not disappear in adult patients either. Abnormalities in Brodmann area 4, which controls behavioral movements, and Brodmann area 8, which together with Brodmann area 6 constitutes the premotor cortex, are likely to be associated with overactive symptoms in ADHD patients. In addition to this, we found changes in both right- and left-brain activation in adult ADHD patients and more extensive changes in right brain activation. This is similar to the changes in the right hemisphere in childhood ADHD patients (Zou and Yang, 2019).

The results in Figure 5 show that there is a correlation between RINs and ANs as well as different areas of different networks. The exchange of information between these areas can be further explained by the fact that brain processing of complex cognitive tasks is not only related to ANs, but RINs are also involved. We also found some weakened connectivity of the auditory area with other networks in adult ADHD patients. For example, the functional connection between Auditory_a and Sensorimotor_i, RFPN_i, and Auditory_i is weakened. This coincides with studies related to ADHD patients showing deficits in the auditory area (Serrallach et al., 2016; Bijlenga et al., 2017). And these findings could not be obtained using the original functional connectivity analysis.

**FIGURE 10:**
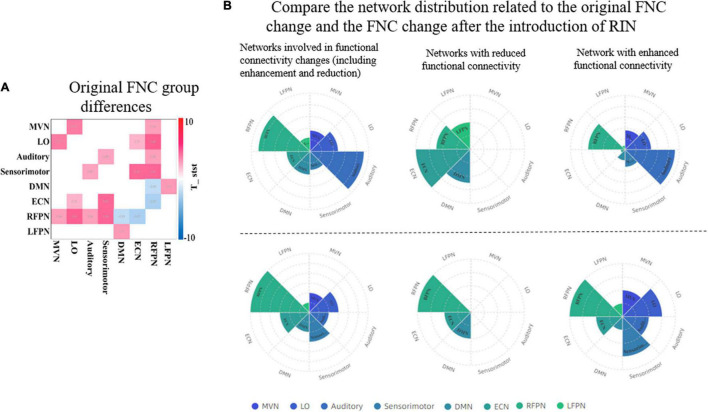
Comparison of the ability of the original network and RINs to detect abnormalities in ADHD patients. **(A)** The group differences of the original FNC. A two-sample *t*-test was used to detect differences between HCs and ADHD patients. Only functional connections with significant differences are shown in the graph (*p* < 0.05, FDR corrected). Red color indicates enhanced functional connectivity in ADHD patients, and blue color indicates weakened functional connectivity. **(B)** The distribution of the networks involved in the FNC group differences detected by the two approaches. The bottom three plots show the FNC change statistics for the original network, and the top three plots show the functional connectivity change statistics after the introduction of RINs. In the top plots, the statistics for each network include the corresponding RIN and AN. For two the left plots, the overall change includes the increase and decrease of functional connectivity; the middle two plots are the networks with weakened functional connectivity; and the two right plots are the networks with enhanced functional connectivity. RIN, relatively inert network; ADHD, attention deficit hyperactivity disorder; FNC, functional network connectivity; HC, healthy control; FDR, false discovery rate.

Across studies, there appear to be significant network disruptions involved in ADHD (Cannon et al., 2011). Therefore, the detection of abnormal connectivity is crucial to further localize areas associated with psychiatric disorders. We further compared the ability to detect abnormal FNCs in ADHD patients using original networks and the introduction of RINs and ANs in Figure 10. In Figure 10A, the FNC changes in ADHD patients are mainly concentrated in the RFPN, while the weakened functional connectivity is reflected in the DMN and ECN. Figure 10B uses pie charts to count the distribution of functional connectivity changes. The two plots on the left depict all changes (including enhancement and weakening). It can be noticed that both results are similar, but more changes in the auditory area can be detected with the introduction of RIN. It is worth noting that the four best-performing features finally filtered in Table 3 are all related to the auditory network, as evidenced by the results in Table 2. Therefore, we have reasons to believe that the auditory changes are real. Moreover, it can also be found in the two plots on the right in subplot (B). Also, for the weakened connections, the original network analysis could not find the weakened LFPN connections. This suggests that the introduction of RINs and ANs may reveal not only the differences analyzed at the original RSN level but also the discovery of additional areas associated with changes in cognitive function in disease. This point is likely to have been overlooked in past studies.

In addition, features constructed based on RINs and ANs (functional connectivity is used in this paper) produce a relatively accurate diagnoses of adult ADHD, with better classification results than those constructed using the original RSN. This effect may be related to the use of the large amount of information present in the RIN. Although it is difficult to avoid the decrease of accuracy in the independent dataset because we did not do more work related to feature engineering (using only functional connectivity), our method still has a greater advantage over the features constructed by the original RSNs. In addition, features based on RINs and ANs, such as dynamic functional connectivity (Sun et al., 2021), dynamic brain fluctuations (Moguilner et al., 2021), or combined with non-image information features (Riaz et al., 2018), are expected to lead to better diagnostic results.

## Conclusion

The exploration of brain RINs has provided new research ideas to study the integration of information between brain areas and disease diagnosis. In this study, we achieved the identification of brain RINs and ANs. Further experiments showed that brain RINs also contain much information about cognitive functions. At the same time, we proposed a new method to achieve diagnosis and study of adult ADHD patients using FNC of RINs and ANs. The experimental results showed that our method well improved the accuracy of disease diagnosis (0.98). This is an initial study of the efficacy of RINs. The ICA-based method we used is also a preliminary attempt. In addition, the eight functional networks we used were not customized for adult ADHD but for the most classical cognitive functional networks in the brain. Therefore, our study is also applicable to the study of other psychiatric disorders, such as bipolar disorder, depression, and Alzheimer’s disease. Due to time constraints, only functional connectivity was used in this study. In the future, we will implement more features such as effective connectivity and dynamic functional connectivity and will continue to explore more functional brain integration and disease diagnostic outcomes in brain RINs.

## Data Availability Statement

The original contributions presented in the study are included in the article/supplementary material, further inquiries can be directed to the corresponding author/s.

## Ethics Statement

The studies involving human participants were reviewed and approved by the Institutional Review Boards at UCLA and the Los Angeles County Department of Mental Health. The patients/participants provided their written informed consent to participate in this study.

## Author Contributions

HZ and WZ contributed to the conception and design of this research. HZ, JD, and YL derived and designed the theoretical and experimental parts in the article. HZ wrote the first draft of the manuscript. HZ, WZ, YS, and LZ participated in the revision, reading, and approval of the manuscript. All authors contributed to the article and approved the submitted version.

## Conflict of Interest

The authors declare that the research was conducted in the absence of any commercial or financial relationships that could be construed as a potential conflict of interest.

## Publisher’s Note

All claims expressed in this article are solely those of the authors and do not necessarily represent those of their affiliated organizations, or those of the publisher, the editors and the reviewers. Any product that may be evaluated in this article, or claim that may be made by its manufacturer, is not guaranteed or endorsed by the publisher.

## Footnotes

1
https://www.fil.ion.ucl.ac.uk/spm/software/spm12/

2 http://icatb.sourceforge.net/, version3.0c.

## References

[B1] Allen E. A. Erhardt E. B. Wei Y. Eichele T. Calhoun V. D. (2012). Capturing inter-subject variability with group independent component analysis of fMRI data: a simulation study. *Neuroimage* 59 4141–4159. 10.1016/j.neuroimage.2011.10.010 22019879 PMC3690335

[B2] Amari S. I. Cichocki A. Yang H. H. (1996). “A new learning algorithm for blind source separation,” in *Proceedings of the Advances in Neural Information Processing Systems 8*, eds Touretzky D. Mozer M. Hasselmo M. (Cambridge MA: MIT Press), 757–763.

[B3] Andersen A. H. Gash D. M. Avison M. J. (1999). Principal component analysis of the dynamic response measured by fMRI: a generalized linear systems framework. *Magn. Reson. Imaging* 17 795–815. 10.1016/S0730-725X(99)00028-4 10402587

[B4] Ashcroft S. Verdolini N. Zaman R. Agius M. (2015). The comorbidity between bipolar disorder and ADHD in a young adult: a focus on impulsivity. *Psychiatr. Danub.* 27(Suppl. 1), 195–197. 26417760

[B5] Assaf M. Jagannathan K. Calhoun V. D. Miller L. Stevens M. C. Sahl R. (2010). Abnormal functional connectivity of default mode sub-networks in autism spectrum disorder patients. *Neuroimage* 53 247–256. 10.1016/j.neuroimage.2010.05.067 20621638 PMC3058935

[B6] Bandettini P. (2010). Processing strategies for time-course data sets in functional MRI of the human brain. *Magn. Reson. Med.* 30 161–173. 10.1002/mrm.1910300204 8366797

[B7] Becke M. Tucha L. Weisbrod M. Aschenbrenner S. Tucha O. Fuermaier A. B. M. (2021). Non-credible symptom report in the clinical evaluation of adult ADHD: development and initial validation of a new validity index embedded in the Conners’ adult ADHD rating scales. *J. Neural Transm.* 128 1045–1063. 10.1007/s00702-021-02318-y 33651237 PMC8295107

[B8] Beckmann C. F. DeLuca M. Devlin J. T. Smith S. M. (2005). Investigations into resting-state connectivity using independent component analysis. *Philos. Trans. R. Soc. B Biol. Sci.* 360 1001–1013. 10.1098/rstb.2005.1634 16087444 PMC1854918

[B9] Bell A. J. Sejnowski T. J. (1995). An information-maximization approach to blind separation and blind deconvolution. *Neural Comput.* 7 1129–1159. 10.1162/neco.1995.7.6.1129 7584893

[B10] Bijlenga D. Tjon-Ka-Jie J. Y. M. Schuijers F. Kooij J. J. S. (2017). Atypical sensory profiles as core features of adult ADHD, irrespective of autistic symptoms. *Eur. Psychiatry* 43 51–57. 10.1016/j.eurpsy.2017.02.481 28371743

[B11] Buitelaar J. Hoogman M. Thompson P. M. Franke B. (2021). White matter microstructure in ADHD: evidence From 2500 individuals from the enigma-ADHD collaboration. *Biol. Psychiatry* 89(Suppl.) S22–S23. 10.1016/j.biopsych.2020.02.244

[B12] Cannon R. Kerson C. Hampshire A. (2011). sLORETA and fMRI detection of medial prefrontal default network anomalies in adult ADHD. *J. Neurother.* 15 358–373.

[B13] Chen J. E. Chang C. Greicius M. D. Glover G. H. (2015). Introducing co-activation pattern metrics to quantify spontaneous brain network dynamics. *Neuroimage* 111 476–488. 10.1016/j.neuroimage.2015.01.057 25662866 PMC4386757

[B14] Chen S. Ross T. J. Wang Z. Myers C. S. Chuang K. S. Heishman S. J. (2008). Group independent component analysis reveals consistent resting-state networks across multiple sessions. *Brain Res.* 1239 141–151. 10.1016/j.brainres.2008.08.028 18789314 PMC2784277

[B15] Chen T. Guestrin C. (2016). “XGBoost: a scalable tree boosting system,” in *Proceedings of the 22nd ACM SIGKDD International Conference on Knowledge Discovery and Data Mining*, (New York, NY: Association for Computing Machinery).

[B16] Cortese S. Adamo N. Del Giovane C. Mohr-Jensen C. Hayes A. J. Carucci S. (2018). Comparative efficacy and tolerability of medications for attention-deficit hyperactivity disorder in children, adolescents, and adults: a systematic review and network meta-analysis. *Lancet Psychiatry* 5 727–738. 10.1016/s2215-0366(18)30269-4 30097390 PMC6109107

[B17] De Blasi B. Caciagli L. Storti S. F. Galovic M. Koepp M. Menegaz G. (2020). Noise removal in resting-state and task fMRI: functional connectivity and activation maps. *J. Neural Eng.* 17:046040. 10.1088/1741-2552/aba5cc 32663803

[B18] dos Santos Siqueira A. Biazoli Junior C. E. Comfort W. E. Rohde L. A. Sato J. R. (2014). Abnormal functional resting-state networks in ADHD: graph theory and pattern recognition analysis of fMRI data. *Biomed. Res. Int.* 2014:380531. 10.1155/2014/380531 25309910 PMC4163359

[B19] Du Y. Fan Y. (2013). Group information guided ICA for fMRI data analysis. *Neuroimage* 69 157–197. 10.1016/j.neuroimage.2012.11.008 23194820

[B20] Duan K. Jiang W. Rootes-Murdy K. Schoenmacker G. H. Arias-Vasquez A. Buitelaar J. K. (2021). Gray matter networks associated with attention and working memory deficit in ADHD across adolescence and adulthood. *Transl. Psychiatry* 11:184. 10.1038/s41398-021-01301-1 33767139 PMC7994833

[B21] Flint C. Cearns M. Opel N. Redlich R. Mehler D. M. Emden D. (2021). Systematic misestimation of machine learning performance in neuroimaging studies of depression. *Neuropsychopharmacology* 46 1510–1517. 33958703 10.1038/s41386-021-01020-7 PMC8209109

[B22] Friston K. J. Jezzard P. Turner R. (2010). Analysis of functional MRI time-series. *Hum. Brain Mapp.* 1 153–171. 10.1002/hbm.460010207

[B23] Himberg J. Hyvarinen A. (2003). “Icasso: software for investigating the reliability of ICA estimates by clustering and visualization,” in *Proceedings of the 2003 IEEE XIII Workshop on Neural Networks for Signal Processing*, (Toulouse: IEEE).

[B24] Hyvarinen A. (1999). Fast and robust fixed-point algorithms for independent component analysis. *IEEE Trans. Neural Netw.* 10 626–634. 18252563 10.1109/72.761722

[B25] Kessler D. Angstadt M. Welsh R. C. Sripada C. (2014). Modality-spanning deficits in attention-deficit/hyperactivity disorder in functional networks, gray matter, and white matter. *J. Neurosci.* 34 16555–16566. 10.1523/JNEUROSCI.3156-14.2014 25505309 PMC4261086

[B26] Kooij S. J. J. Bejerot S. Blackwell A. Caci H. Casas-Brugué M. Carpentier P. J. (2010). European consensus statement on diagnosis and treatment of adult ADHD: the European network adult ADHD. *BMC Psychiatry* 10:67. 10.1186/1471-244X-10-67 20815868 PMC2942810

[B27] Li Y.-O. Adalı T. Calhoun V. D. (2007). Estimating the number of independent components for functional magnetic resonance imaging data. *Hum. Brain Mapp.* 28 1251–1266. 10.1002/hbm.20359 17274023 PMC6871474

[B28] Lin Q. H. Liu J. Zheng Y. R. Liang H. Calhoun V. D. (2010). Semiblind spatial ICA of fMRI using spatial constraints. *Hum. Brain Mapp.* 31 1076–1088. 10.1002/hbm.20919 20017117 PMC2891131

[B29] Mckeown M. J. Makeig S. Brown G. G. Jung T. P. Kindermann S. S. And A. (1998). Analysis of fMRI data by blind separation into independent spatial components. *Hum. Brain Mapp.* 6 160–188. 10.1002/(SICI)1097-019319986:3<160::AID-HBM5<3.0.CO;2-1 9673671 PMC6873377

[B30] Moguilner S. García A. M. Perl Y. S. Tagliazucchi E. Piguet O. Kumfor F. (2021). Dynamic brain fluctuations outperform connectivity measures and mirror pathophysiological profiles across dementia subtypes: a multicenter study. *Neuroimage* 225:117522. 33144220 10.1016/j.neuroimage.2020.117522 PMC7832160

[B31] Nickerson L. D. Smith S. M. Öngür D. Beckmann C. F. (2017). Using dual regression to investigate network shape and amplitude in functional connectivity analyses. *Front. Neurosci.* 11:115. 10.3389/fnins.2017.00115 28348512 PMC5346569

[B32] Poldrack R. A. Congdon E. Triplett W. Gorgolewski K. J. Karlsgodt K. H. Mumford J. A. (2016). A phenome-wide examination of neural and cognitive function. *Sci. Data* 3:160110. 10.1038/sdata.2016.110 27922632 PMC5139672

[B33] Power J. D. Cohen A. L. Nelson S. M. Wig G. S. Petersen S. E. (2011). Functional network organization of the human brain. *Neuron* 72 665–678. 10.1016/j.neuron.2011.09.006 22099467 PMC3222858

[B34] Qian X. Castellanos F. X. Uddin L. Q. Loo B. R. Y. Liu S. Koh H. L. (2019). Large-scale brain functional network topology disruptions underlie symptom heterogeneity in children with attention-deficit/hyperactivity disorder. *Neuroimage Clin.* 21:101600. 10.1016/j.nicl.2018.11.010 30472167 PMC6411599

[B35] Riaz A. Asad M. Alonso E. Slabaugh G. (2018). Fusion of fMRI and non-imaging data for ADHD classification. *Comput. Med. Imaging Graph* 65 115–128. 29137838 10.1016/j.compmedimag.2017.10.002

[B36] Roshannia S. Akhlaghi Z. Kordestani-Moghadam P. (2021). A review of cognitive disorders in attention deficit hyperactivity disorder with emphasis on executive functions and brain structures. *Int. Clin. Neurosci. J.* 8 60–66. 10.34172/icnj.2021.14

[B37] Rovira P. Demontis D. Sánchez-Mora C. Zayats T. Klein M. Mota N. (2020). Shared genetic background between children and adults with attention deficit/hyperactivity disorder. *Neuropsychopharmacology* 45 1–12. 10.1038/s41386-020-0664-5 32279069 PMC7419307

[B38] Schiweck C. Arteaga-Henriquez G. Aichholzer M. Thanarajah S. E. Reif A. (2021). Comorbidity of ADHD and adult bipolar disorder: a systematic review and meta-analysis. *Neurosci. Biobehav. Rev.* 124 100–123. 10.1016/j.neubiorev.2021.01.017 33515607

[B39] Schneider M. Retz W. Coogan A. Thome J. Rösler M. (2006). Anatomical and functional brain imaging in adult attention-deficit/hyperactivity disorder (ADHD)—a neurological view. *Eur. Arch. Psychiatr. Clin. Neurosci.* 256 i32–i41. 10.1007/s00406-006-1005-3 16977550

[B40] Serrallach B. Groß C. Bernhofs V. Engelmann D. Benner J. Gündert N. (2016). Neural biomarkers for dyslexia, ADHD, and ADD in the auditory cortex of children. *Front. Neurosci.* 10:324. 10.3389/fnins.2016.00324 27471442 PMC4945653

[B41] Shaw P. Malek M. Watson B. Greenstein D. Rossi P. D. Sharp W. (2013). Trajectories of cerebral cortical development in childhood and adolescence and adult attention-deficit/hyperactivity disorder. *Biol. Psychiatry* 74 599–606. 10.1016/j.biopsych.2013.04.007 23726514 PMC5922431

[B42] Slavakis K. Salsabilian S. Wack D. S. Muldoon S. F. Baidoo-Williams H. E. Vettel J. M. (2018). “Clustering brain-network time series by Riemannian geometry,” in *Proceedings of the IEEE Transactions on Signal and Information Processing over Networks*, Vol. 4, (Piscataway, NJ: IEEE), 519–533. 10.1109/TSIPN.2017.2774504

[B43] Smith S. M. Hyvrinen A. Varoquaux G. Miller K. L. Beckmann C. F. (2014). Group-PCA for very large fMRI datasets. *Neuroimage* 101 738–749. 10.1016/j.neuroimage.2014.07.051 25094018 PMC4289914

[B44] Smith S. T. Cox J. Mowle E. N. Edens J. F. (2017). Intentional inattention: detecting feigned attention-deficit/hyperactivity disorder on the personality assessment Inventory. *Psychol. Assess.* 29 1447–1457. 10.1037/pas0000435 29227126

[B45] Smolders A. Valente G. Martino D. F. Staeren N. Scheunders P. Sijbers J. (2007). “Spatio-temporal fuzzy clustering of fMRI timeseries,” in *Proceedings of the Joint Annual Meeting ISMRM-ESMRMB*, Berlin.

[B46] Spencer T. J. Biederman J. Mick E. (2007). Attention-deficit/hyperactivity disorder: diagnosis, lifespan, comorbidities, and neurobiology. *J. Pediatr. Psychol.* 32:631. 10.1016/j.ambp.2006.07.006 17556405

[B47] Sudre G. Szekely E. Sharp W. Kasparek S. Shaw P. (2017). Multimodal mapping of the brain’s functional connectivity and the adult outcome of attention deficit hyperactivity disorder. *Proc. Natl. Acad. Sci. U.S.A.* 114:11787. 10.1073/pnas.1705229114 29078281 PMC5676882

[B48] Sun Y. Lan Z. Xue S.-W. Zhao L. Xiao Y. Kuai C. (2021). Brain state-dependent dynamic functional connectivity patterns in attention-deficit/hyperactivity disorder. *J. Psychiatr. Res.* 138 569–575. 33991995 10.1016/j.jpsychires.2021.05.010

[B49] Suskauer S. J. Simmonds D. J. Caffo B. S. Denckla M. B. Pekar J. J. Mostofsky S. H. (2008). fMRI of intrasubject variability in ADHD: anomalous premotor activity with prefrontal compensation. *J. Am. Acad. Child Adolesc. Psychiatry* 47 1141–1150. 18724253 10.1097/CHI.0b013e3181825b1f PMC3932630

[B50] Versace A. Jones N. P. Joseph H. M. Lindstrom R. A. Wilson T. K. Lima Santos J. P. (2021). White matter abnormalities associated with ADHD outcomes in adulthood. *Mol. Psychiatry* 1–11. 10.1038/s41380-021-01153-7 34035475 PMC8613296

[B51] Wei L. Rajapakse J. C. (2005). “Approach and applications of constrained ICA,” in *Proceedings of the IEEE Transactions on Neural Networks*, Vol. 16, (Piscataway, NJ: IEEE), 203. 10.1109/TNN.2004.836795 15732400

[B52] Yan C. G. Wang X. D. Zuo X. N. Zang Y. F. (2016). DPABI: data processing & analysis for (resting-state) brain imaging. *Neuroinformatics* 14 339–351. 27075850 10.1007/s12021-016-9299-4

[B53] Zou H. Yang J. (2019). Temporal variability-based functional brain lateralization study in ADHD. *J. Atten. Disord.* 25 839–847. 10.1177/1087054719859074 31268386

